# Participation, retention, and associated factors of women in a prospective multicenter study on *Chlamydia trachomatis* infections (FemCure)

**DOI:** 10.1371/journal.pone.0230413

**Published:** 2020-03-18

**Authors:** Nicole H. T. M. Dukers–Muijrers, Titia Heijman, Hannelore M. Götz, Patricia Zaandam, Juliën Wijers, Jeanine Leenen, Geneviève van Liere, Jeanne Heil, Stephanie Brinkhues, Astrid Wielemaker, Maarten F. Schim van der Loeff, Petra F. G. Wolffs, Sylvia M. Bruisten, Mieke Steenbakkers, Arjan A. Hogewoning, Henry J. de Vries, Christian J. P. A. Hoebe

**Affiliations:** 1 Department of Sexual Health, Infectious Diseases, and Environmental Health, South Limburg Public Health Service, Heerlen, The Netherlands; 2 Department of Social Medicine and Medical Microbiology, Care and Public Health Research Institute (CAPHRI), Maastricht University, Maastricht, The Netherlands; 3 Department of Infectious Diseases, Public Health Service of Amsterdam (GGD Amsterdam), Amsterdam, The Netherlands; 4 Department of Public Health, Sexual Health Centre, Public Health Service Rotterdam-Rijnmond, Rotterdam, The Netherlands; 5 National Institute of Public Health and the Environment (RIVM), Epidemiology and Surveillance Unit, Centre for Infectious Disease Control, Bilthoven, The Netherlands; 6 Department of Public Health, Erasmus MC—University Medical Center Rotterdam, Rotterdam, The Netherlands; 7 Department of Dermatology, Amsterdam UMC, University of Amsterdam, Amsterdam Institute for Infection and Immunity (AI&II), Location Academic Medical Centre, Amsterdam, The Netherlands; Xavier Bichat Medical School, INSERM-CNRS - Université Paris Diderot, FRANCE

## Abstract

Prospective studies are key study designs when attempting to unravel health mechanisms that are widely applicable. Understanding the internal validity of a prospective study is essential to judge a study’s quality. Moreover, insights in possible sampling bias and the external validity of a prospective study are useful to judge the applicability of a study’s findings. We evaluated participation, retention, and associated factors of women in a multicenter prospective cohort (FemCure) to understand the study’s validity.*Chlamydia trachomatis* (CT) infected adult women, negative for HIV, syphilis, and *Neisseria gonorrhoeae* were eligible to be preselected and included at three sexually transmitted infection (STI) clinics in the Netherlands (2016–2017). The planned follow-up for participants was 3 months, with two weekly rectal and vaginal CT self-sampling and online questionnaires administered at home and at the clinic. We calculated the proportions of preselected, included, and retained (completed follow-up) women. Associations with non-preselection, noninclusion, and non-retention (called attrition) were assessed (logistic and Cox regression).Among the 4,916 women, 1,763 (35.9%) were preselected, of whom 560 (31.8%) were included. The study population had diverse baseline characteristics: study site, migration background, high education, and no STI history were associated with non-preselection and noninclusion. Retention was 76.3% (n = 427). Attrition was 10.71/100 person/month (95% confidence interval 9.97, 12.69) and was associated with young age and low education. In an outpatient clinical setting, it proved feasible to include and retain women in an intensive prospective cohort. External validity was limited as the study population was not representative (sampling bias), but this did not affect the internal validity. Selective attrition, however (potential selection bias), should be accounted for when interpreting the study results.

## Introduction

Prospective studies are key study designs for assessing the risks of certain determinants in acquiring a specific disease in an attempt to unravel health mechanisms that are widely applicable [1,2]. Women’s participation in prospective studies is fundamental to understanding their health mechanisms [3]. Despite a continued emphasis by regulatory bodies, health institutions, research funding organizations, and scientific journals, the inclusion of women in research is not easy [4,5]. Significant benefits for prospective research in women can be reaped by sharing best practices on inclusion and retention.

First and foremost, a prospective study should have high internal validity to generate unconfounded insights [1]. Understanding a study’s internal validity is essential to judging its quality. Internal validity may be compromised when it is difficult to include sufficient participants. Low study power may result in associations that are spurious (“false”), imprecise (with wide confidence intervals), or missed altogether [6,7], and it becomes difficult to replicate the study. Similar threats to internal validity arise when insufficient participants are retained, i.e., when a large portion of included women is lost to follow-up or withdraws from the study (both called attrition). When attrition disproportionally affects a subset of the study population, it may lead to selection bias and an underestimation or an overestimation of the associations [1,8].

When a prospective study has high internal validity, the risks obtained may be applicable to a broad population of women and the study population does not need to have high external validity, i.e., be a representative sample [9–11]. Usually, representative frequency distributions are derived from other types of research, i.e., population-based surveys [9]. In practice, it has proven to be logistically difficult and cost ineffective to include and follow a representative sample in a study that, first and foremost, is designed to achieve high internal validity [12]. Still, insights in possible sampling bias and the external validity of a prospective study are useful to judge the applicability of a study’s findings. Internal and external validity reporting has improved with the STROBE and CONSORT guidelines [13] and is increasingly encouraged in the scientific journals [14].

Here, we report on the FemCure study, the first prospective research that systematically addresses rectal *Chlamydia trachomatis* (CT) in women [15]. FemCure included 560 women from three Dutch STI clinics and followed them for 3 months with systematic rectal and vaginal CT assessment. Chlamydia infection is the most commonly reported treatable bacterial sexually transmitted infection (STI) in high-income countries [16,17]. CT disproportionally affects women in terms of its occurrence and burden of sequelae, i.e., pelvic inflammatory disease, ectopic pregnancy, and tubal infertility [18]. The main treatments are doxycycline and azithromycin [19,20], although, the efficacy of azithromycin in the treatment of CT is debatable [21]. FemCure was set up to improve our understanding of posttreatment rectal and vaginal CT detection in women, by assessing risks due to sexual exposure (horizontal transmission) or exposure from adjacent body sites (self-infection), and by assessing the role of possibly suboptimal treatment effectiveness [15].

Here, we describe the multifaceted strategies that were implemented to include and retain a group of women attending STI clinics in a prospective cohort study. Insights in potential sampling bias and external validity of the FemCure study population as well as potential selection bias of the retained sample (internal validity) are presented by analyzing the factors associated with nonparticipation and attrition.

## Materials and methods

### Ethics approval and consent to participate

All participants provided written informed consent. This study was approved by the Medical Ethical Review Committee from the Maastricht University Medical Centre, Maastricht Netherlands (NL51358.068.15/METC153020, 20-01-2016) and monitored by the Clinical Trial Centre Maastricht. ClinicalTrials.gov Identifier: NCT02694497.

### Study design

#### STI clinic setting for the study

**Fig 1:**
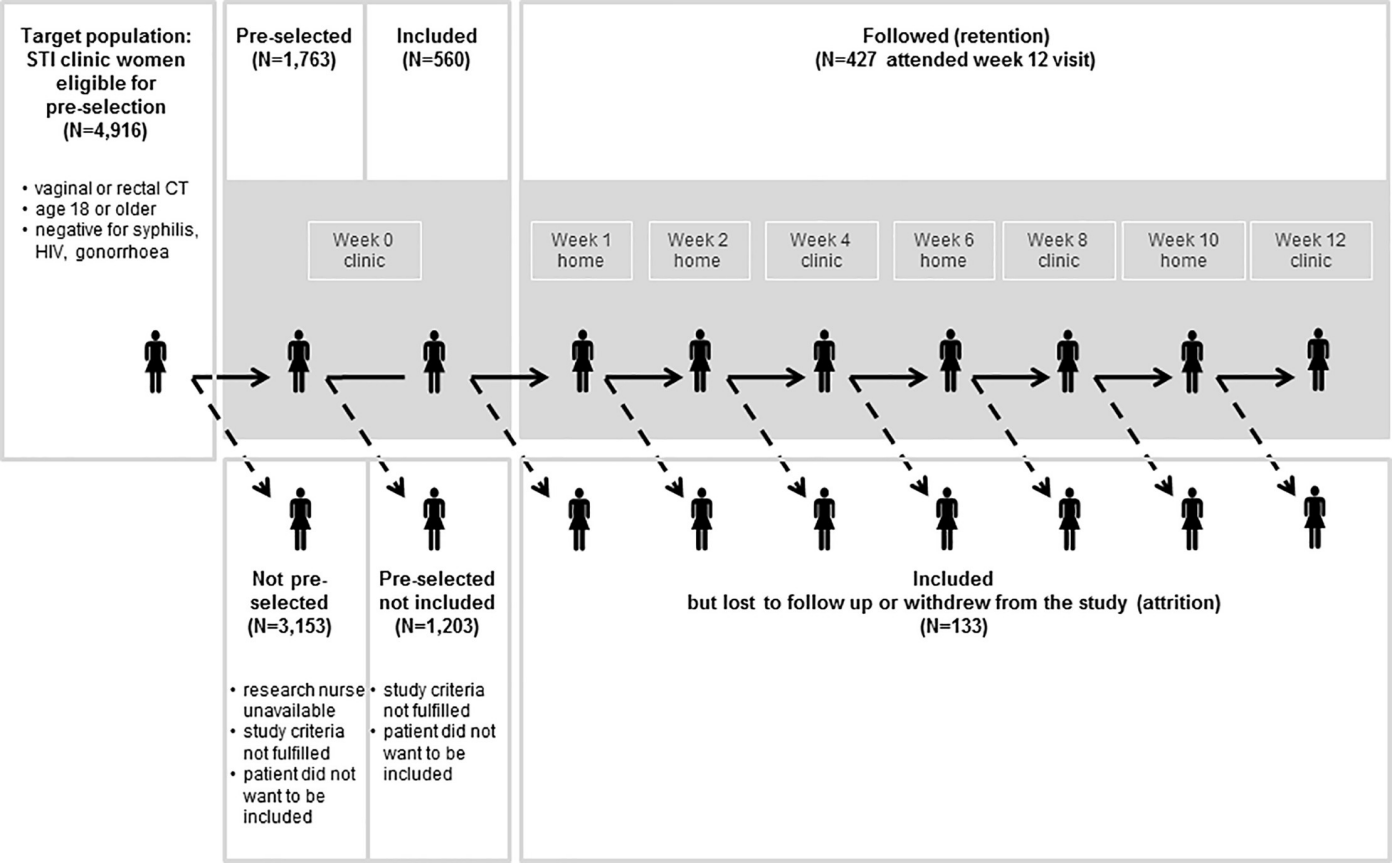
Design of the prospective cohort study, FemCure.

The study population originated from the STI clinics of the Public Health Services in South Limburg, Rotterdam, and Amsterdam, which represent almost half of all STI clinic consultations in the Netherlands. Clinics apply the same care procedures [19] but vary in their annual number of clients (6,000–45,000) and urbanization degree (rural to highly urban) [22]. According to European guidelines [19], all women were tested for vaginal CT, and they were also rectally tested, when reporting anal sex or anal symptoms. Women who tested positive for rectal infection were treated with a seven-day course of oral doxycycline 100 mg twice daily [19]. Women who tested positive for vaginal infection, and who either tested negative for rectal infection or did not undergo the rectal test, received a 1 g single azithromycin oral dose. The first doxycycline and azithromycin doses were directly observed. In this context, we set up a cohort study with 3 months of follow-up (Fig 1) [15].

#### Target population

The target population of FemCure were women, who were 18 years or older, diagnosed with a vaginal or rectal CT infection during the inclusion period (April 2016 until September 2017), and negative for HIV, syphilis, and *Neisseria gonorrhoeae* (NG). FemCure aimed to have at least 400 women with complete follow-up, for sufficient power for the planned analyses [15]. To maximize participation, multifaceted strategies focused on helpful themes, identified previously [23–31], were simultaneously implemented (Table 1).

**Table 1:** Themes and strategies (derived from previous studies 23–31) employed in the prospective multicenter FemCure study, aimed to maximize women’s participation.

Themes	Strategies
Allow long preparation phase	• Acquire funding, acquire institutional review board approval, install logistical requirements, install management systems, and train all staff
Easy access to the study population	• Low-threshold STI clinics ensured a continuous availability of chlamydia-infected adult women.
	• Before inclusion of potential participants, eligibility was prechecked by the clinic nurses.
Clear study information for patients	• Study information was provided to women at least a day before the inclusion visit, to give time for an informed decision.
	• A public website was launched with study information and procedures (www.FemCure.nl).
	• The research nurses were available to explain the study during face-to-face contact.
Study theme relevant for the patient	• The study addressed the health problem (vaginal and/or rectal chlamydia) that patients had.
Easy data collection	• Women could participate from their homes and in the vicinity of their homes, i.e., at the STI clinic where they initially went for their STI testing.
	• For women with transportation constraints, financial compensation was provided to cover transport costs.
	• Kits for self-sampling and online questionnaires enabled data collection at the patient’s own pace in the privacy of their home.
	• Participants received study material, instructions, and a personal booklet with all study appointments.
	• At each STI clinic study visit, the nurse provided face-to-face clarification when needed.
Familiar and close-contact environment	• The study setting and procedures were fully integrated into a clinical setting, which the participants were already familiar with.
	• Follow-up visits of the participant were scheduled as much as possible with the same nurse who included the woman.
	• Participants could be contacted by (or herself contact) the research nurse in case of questions. A study-specific mobile telephone and email address were used and monitored several times each day to ensure timely responses to participants.
	• Short text messages were used to remind the women to complete the questionnaire and take the self-samples. Women who did not respond were contacted multiple times.
Incentives for patients	• At each clinic visit, the participants received a small 10-euro gift certificate.
	At the last clinic visit, each participant was offered oral, vaginal, and rectal CT/NG testing, and treatment when needed, free of charge.
High-functioning staff: research nurses (n = 18)	• Handled the inclusion and follow-up study-visits at weeks 0, 4, 8, and 12 at the three study sites.
	• Highly experienced in STI care, available part-time for study duties and part-time for routine clinic duties.
	• Fully trained for the study procedures, online questionnaires, and for good clinical practices.
	• Sufficient time was allocated to handle the patient visits.
	• Daily local supervision by dedicated site coordinators and available back-office staff to provide support in case of questions.
	• Interim site meetings were organized to discuss inclusion, retention, facilitators, and barriers.
High-functioning staff: logistical staff (n = 6)	• Handled retention activities using a well-built data management system. Reminder text messages were sent daily. Additional contact attempts were via phone and via email. Research nurses were contacted when needed.
	• Back office. A special email address and a secure internet environment were created to allow safe communication between staff.
	• Regular transport of collected stored samples from the study sites to the coordinating laboratory (Maastricht).
	• Gave feedback on progress in order to optimize procedures, inclusion, and retention.
	• Fully trained in the study procedures, good clinical practice, and management activities.
High-functioning staff: laboratory staff (n = 5)	• Handled sample registration, storage, laboratory testing, and provision of information to the data manager.
High-functioning staff: project coordinator (n = 1) and site coordinators (n = 3)	• Monitored inclusion and retention throughout the study to ensure that targeted goals were met.
	• Supervised the overall processes in conjunction with the site coordinators and also handled institutional review board and study oversight.

CT: *Chlamydia trachomatis*; NG: *Neisseria gonorrhoeae*; STI: sexually transmitted infections

#### Who was preselected for inclusion?

Study information was provided to the women at least a day before the inclusion visit (the treatment consultation), to give them time to make an informed decision. Therefore, a standardized text and link to the study’s website were communicated via the routine channels that clinics use for contacting patients (i.e., online and by text message). Women from the target population could be preselected at the treatment consultation when the following applied: (a) a research nurse was available at that moment to handle inclusion (the predefined research capacity for handling study visits was four to six nurses per clinic; nurses were required to be available part-time for study duties, a priori restricting the number of patients who could be preselected); (b) study criteria applied (no recent antibiotic use, not pregnant, received study information at least 1 day before participation, understand the Dutch language, able to complete the study [e.g., not going abroad during the study, living close to the clinic]); (c) the patient was willing to, at that moment, be fully informed about the study and, when accepting inclusion, to comply with the study procedures; (d) the patient was willing to be transferred to a research nurse for possible inclusion in case if the patient was not already managed by a research nurse.

#### Who was included in the study?

For preselected women, the research nurse provided information and checked study criteria. A calendar was used to plan follow-up clinic visits. When a patient accepted the study procedures, providing written informed consent, the research nurse handled inclusion and provided treatment. Reasons for noninclusion were scored.

#### Who was followed after inclusion?

All included women were followed, but they could withdraw from the study or become lost-to-follow-up (both: attrition). Reasons for study withdrawal were recorded.

#### Data collection

Clinical data (such as baseline characteristics of the target population) originated from the electronic clinic patient registries. Participants in the study self-collected rectal and vaginal samples for CT testing and completed structured online self-administered questionnaires (on antibiotic use, symptoms, and sexual behavior) at inclusion (week 0), and at weeks 4, 8, and 12 at the STI clinic, and at weeks 1, 2, 6, and 10 at home (Fig 1). A test package with clear instructions was provided for self-collection of samples at home. Week 12 data also included a study evaluation.

### Statistical analyses

#### Aim and outcomes

For the current analysis, we aimed to elucidate aspects of internal and external validity of the FemCure study. We analyzed participation (preselection, inclusion) and retention, and factors (patient characteristics) associated with nonparticipation (non-preselection, noninclusion) and attrition.

#### Participation

Preselection was the proportion of women preselected from the target population. Inclusion was the proportion included from the preselected women and the proportion of women included from the target population.

#### Retention

Retention was the proportion of included women who attended the week 12 visit. Kaplan–Meier techniques with 95% confidence intervals (95% CI) were used to show retention over time.

#### Non-participation

Non-preselection and noninclusion were assessed as proportions.

#### Attrition

Attrition, i.e., withdrawal or lost-to-follow-up, was assessed as a rate (per 100 person months [PM]) and 95% CI.

#### Factors evaluated

Characteristics at baseline were: study site (South Limburg, Rotterdam, Amsterdam), age (18–20, 21–23, ≥24 years), migration background (Western, Asian/Turkish, African, Latin America, unknown), education (low, middle, high, unknown), STI history (yes, no, unknown), diagnosed CT (vaginal [and rectal untested], vaginal [and rectal negative], vaginal and rectal, rectal [and vaginal negative]), number of sex partners in the past 6 months (0 or 1, 2 or 3, ≥ 4, unknown). The educational level was measured as current education or highest educational level completed and was categorized into three categories: lower educated (pre-vocational secondary education, secondary vocational education), medium educated (senior general secondary education, pre-university education) and higher educated (higher professional education, university education).

#### Associations with nonparticipation

To examine potential selective participation and explore sampling bias, associated factors for non-preselection in the target population, and for noninclusion among preselected women, were assessed by univariate and multivariate logistic regression analyses using a stepwise backward approach, and expressed by odds ratios (OR) and 95% CI. Associated factors for noninclusion were also assessed in the target population to explore the representativeness of the study population.

#### Associations with attrition

To examine potential selective attrition and explore selection bias, factors associated with attrition risk were assessed using univariate and multivariate Cox regression analyses and expressed by hazard ratios (HR) and 95% CI.

#### Descriptive statistics

Descriptive statistics were used for the distribution of the patient characteristics and for the reasons for noninclusion or attrition. For the retained women, we described the number of missing samples and questionnaires, and the women’s week 12 study evaluation responses.

#### Statistical software

We used the IBM SPSS Statistics 21 and Stata StataCorp 15.

## Results

### Target population

In the study period, 4,916 women who were eligible for preselection came to the participating clinics (Table 2).

**Table 2:** Baseline characteristics distributions in the target population, (not) preselected women, (not) included women, and women retained in the study, FemCure.

	Target population (N = 4,916) ^a^			Preselected women (N = 1,763)		
	Total N = 4,916	Not preselected N = 3,153	Preselected N = 1,763	Preselected not included N = 1,203	Included N = 560	Included and retained N = 427
	n (%)	n (%)	n (%)	n (%)	n (%)	n (%)
Study site						
South Limburg	974 (19.8)	504 (16.0)	470 (26.7)	271 (22.5)	199 (35.5)	154 (36.1)
Amsterdam	2,875 (58.5)	2,099 (66.6)	776 (44.0)	579 (48.1)	197 (35.2)	156 (36.5)
Rotterdam	1,067 (21.7)	550 (17.4)	517 (29.3)	353 (29.3)	164 (29.3)	117 (27.4)
Age (in years)						
18–20	1,382 (28.1)	853 (27.1)	529 (30.0)	367 (30.5)	162 (28.9)	105 (24.6)
21–23	1,935 (39.4)	1,221 (38.7)	714 (40.5)	510 (42.4)	204 (36.4)	161 (37.7)
≥24	1,599 (32.5)	1,079 (34.2)	520 (29.5)	326 (27.1)	194 (34.6)	161 (37.7)
Background						
Western	3,962 (80.6)	2,476 (78.5)	1,486 (84.3)	969 (80.5)	517 (92.3)	400 (93.7)
Asian/Turkish migrant	186 (3.8)	123 (3.9)	63 (3.6)	48 (4.0)	15 (2.7)	10 (2.3)
African migrant	222 (4.5)	149 (4.7)	73 (4.1)	61 (5.1)	12 (2.1)	10 (2.3)
Latin American migrant	545 (11.1)	405 (12.8)	140 (7.9)	124 (10.3)	16 (2.9)	7 (1.6)
Unknown ^b^	1 (0.0)	0 (0)	1 (0.1)	1 (0.1)	0 (0)	0 (0)
Education						
Low	1,513 (30.8)	941 (29.8)	572 (32.4)	369 (30.7)	203 (36.3)	135 (31.6)
Middle	661 (13.4)	327 (10.4)	334 (18.9)	127 (10.6)	207 (37.0)	160 (37.5)
High	2,533 (51.5)	1,705 (54.1)	828 (47.0)	679 (56.4)	149 (26.6)	131 (30.7)
Unknown	209 (4.3)	180 (5.7)	29 (1.6)	28 (2.3)	1 (0.2)	1 (0.2)
STI history						
Yes	743 (15.1)	403 (12.8)	340 (19.3)	175 (14.5)	165 (29.5)	124 (29.0)
No	3,031 (61.7)	2,017 (64.0)	1014 (57.5)	626 (52.0)	388 (69.3)	297 (69.6)
Unknown ^b^	1,142 (23.2)	733 (23.2)	409 (23.2)	402 (33.4)	7 (1.3)	6 (1.4)
CT diagnosis						
vCT (rCT not tested)	2,880 (58.6)	1,760 (55.8)	1,120 (63.5)	769 (63.9)	351 (62.7)	264 (61.8)
vCT (rCT negative)	273 (5.6)	188 (6.0)	85 (4.8)	60 (5.0)	25 (4.5)	19 (4.4)
vCT & rCT	1,458 (29.7)	991 (31.4)	467 (26.5)	312 (25.9)	155 (27.7)	118 (27.6)
rCT (vCT negative)	305 (6.2)	214 (6.8)	91 (5.2)	62 (5.2)	29 (5.2)	26 (6.1)
No. of sex partners						
0 or 1	1,158 (23.6)	762 (24.2)	396 (22.5)	270 (22.4)	126 (22.5)	95 (22.2)
2 or 3	2,242 (45.6)	1,388 (44.0)	854 (48.4)	587 (48.8)	267 (47.7)	194 (45.4)
≥ 4	1,486 (30.2)	987 (31.3)	499 (28.3)	334 (27.8)	165 (29.5)	136 (31.9)
unknown ^b^	30 (0.6)	16 (0.5)	14 (0.8)	12 (1.0)	2 (0.4)	2 (0.5)

rCT: rectal *Chlamydia trachomatis*, vCT: vaginal *Chlamydia trachomatis*

^a^ Women 18 years or older with vaginal or rectal CT and negative for HIV, syphilis, and *Neisseria gonorrhoeae*, during the study inclusion period: Apr. 2016–Sept. 2017

^b^ missing information

### Participation

#### Preselection

Among the 4,916 women, 1,763 (35.9%) were preselected.

#### Inclusion

Among the preselected women, 560 (31.8%) were included (Table 2). Included women comprised 11.4% of the target population. Table 2 presents the baseline characteristics distribution in different steps from the target population to the included population. Among the included women, 28.9% were young (18–20 years), 36.3% had a low educational level, and 7.7% had a non-Western migration background.

### Retention

**Fig 2:**
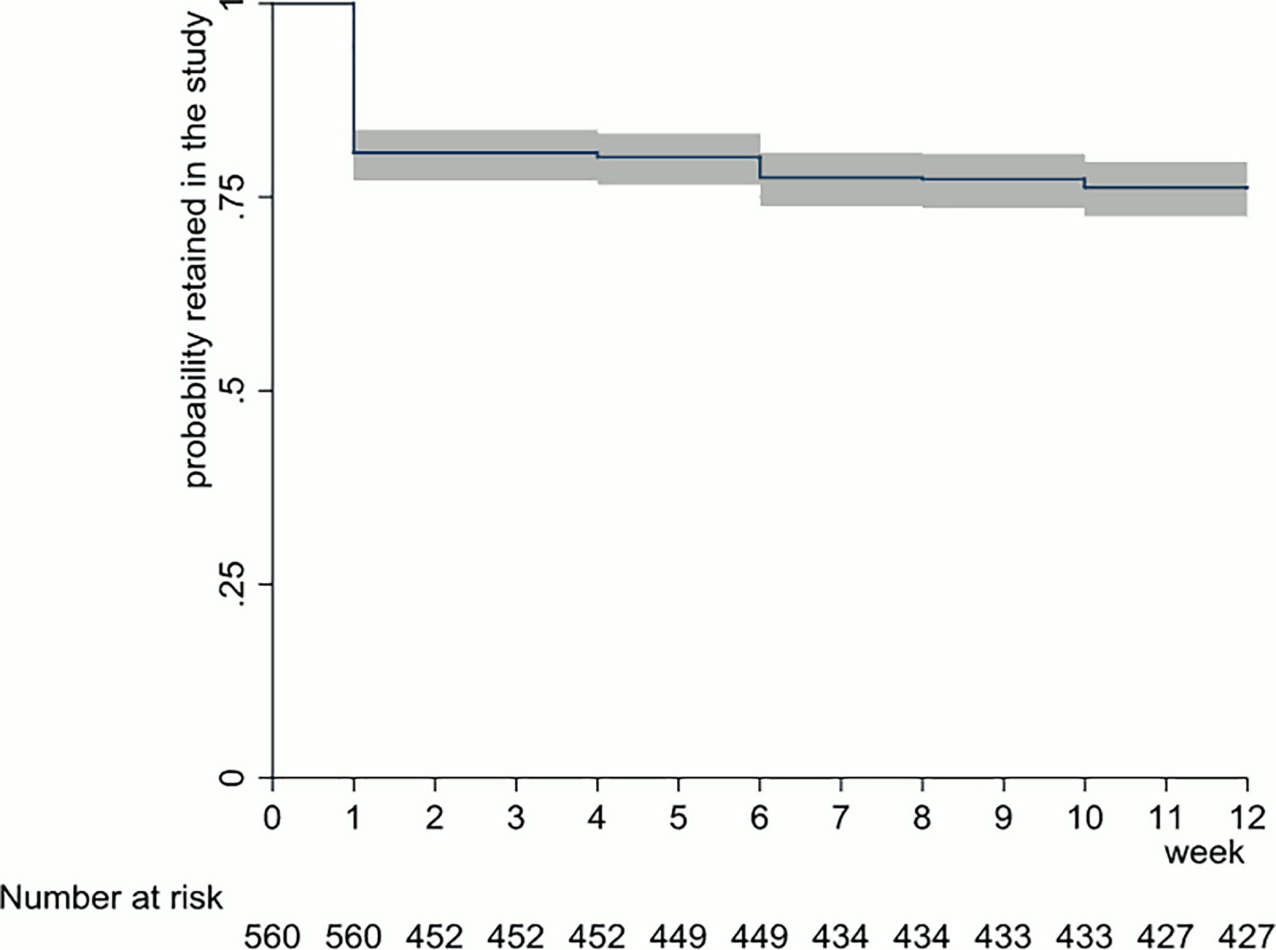
Kaplan–Meier survival function (line) with 95% confidence intervals (grey area) on the probability of being retained, and the number of women retained (“Number at Risk”) in the FemCure study.

Among the included women, 427 (76.2%) were retained (Fig 2, Table 2). Together, these 427 women collected data 3,416 scheduled times. By the seventeenth scheduled time (0.5%, 15 women), samples and questionnaire data were not provided in 3 of these moments. Further, at 14 moments, questionnaire data were not provided.

### Nonparticipation

#### Non-preselection

Among the 4,916 women in the target population, 3,153 (64.1%) were not preselected.

#### Noninclusion

Among the 1,763 preselected women, 1,203 (68.2%) were not included.

Reasons for noninclusion were related to study criteria in 556 of the not included women, i.e., unable to attend clinic visits as required (n = 450), insufficient understanding of the Dutch language (n = 41), not accepting directly observed treatment (n = 48), or sample collection at home (n = 17). A total of 590 women were not included due to patient-related reasons (too much expected effort or time). There were 57 patients who declined due to other reasons or did not provide a reason.

### Attrition

Among the 560 included women, 133 withdrew from the study or were lost-to-follow-up. The attrition rate was 10.71/100 PM (95% CI 9.97, 12.69). Women who were lost to follow up (n = 66) did not differ in their baseline characteristics (*P* >0.05 by two-sided chi-squared testing) compared to women who withdrew (n = 67). Women who withdrew stated they were incapable or unwilling to invest further time in the study (n = 43) or had other reasons (n = 24). Reasons for stopping sample provision were unknown when women were lost to follow up as they did not respond to the text messages or telephone calls of the research nurse.

### Factors associated with nonparticipation

#### Associated factors for non-preselection from the target population

In the multivariate analyses (Table 3), factors independently associated with non-preselection were study site Amsterdam (compared to South Limburg), non-Western migration background, and no STI history. A medium educational level (compared to a low educational level) was inversely associated.

**Table 3:** Odds ratios for the association between characteristics and non-preselection among STI clinic women target population, and noninclusion among preselected women, and noninclusion among the target population, FemCure.

	Non-preselection from target population ^a^				Noninclusion from preselected women ^a^				Noninclusion from target population ^a^			
	OR (95% CI)	*P*	aOR (95% CI)	*P*	OR (95% CI)	p	aOR (95% CI)	*P*	OR (95% CI)	*P*	aOR (95% CI)	*P*
Study site		<0.01		<0.01		<0.01		<0.01		<0.01		<0.01
South Limburg	1		1		1		1		1		1	
Amsterdam	2.52 (2.17,2.93)		3.70 (3.06,4.47)		2.16 (1.69,2.76)		4.45 (3.20,6.21)		3.49 (2.82,4.32)		7.66 (5.90,9.95)	
Rotterdam	0.99 (0.83,1.18)		0.92 (0.76,1.11)		1.58 (1.22,2.05)		1.37 (0.95,1.98)		1.41 (1.13,1.78)		1.30 (0.98,1.73)	
Age (in years)		<0.01				<0.01		<0.01		0.30		
18–20	0.78 (0.67,0.90)				1.35 (1.04,1.74)		3.65 (2.53,5.27)		1.04 (0.83,1.30)			
21–23	0.82 (0.72,0.95)				1.49 (1.17,1.89)		3.10 (2.25,4.27)		1.17 (0.95,1.44)			
≥24	1				1		1		1			
Background		<0.01		<0.01		<0.01		<0.01		<0.01		<0.01
Western	1		1		1		1		1		1	
Non west. Migrant	1.47 (1.26,1.72)		1.32 (1.12,1.57)		2.89 (2.05,4.07)		3.03 (2.00,4.58)		3.18 (2.31,4.37)		2.84 (2.00,4.04)	
Unknown ^b^												
Education		<0.01		<0.01		<0.01		<0.01		<0.01		<0.01
Low	1		1		1		1		1		1	
Middle	0.60 (0.50,0.72)		0.52 (0.43,0.64)		0.34 (0.26,0.45)		0.28 (0.19,0.40)		0.34 (0.27,0.42)		0.32 (0.25,0.42)	
High	1.25 (1.10,1.43)		1.01 (0.87,1.17)		2.51 (1.96,3.21)		3.09 (2.24,4.45)		2.48 (2.00,3.10)		2.20 (1.71,2.83)	
Unknown ^b^	na											
STI history		<0.01		<0.01		<0.01		<0.01		<0.01		<0.01
Yes	1		1		1		1		1		1	
No	1.68 (1.43,1.98)		1.24 (1.04,1.48)		1.52 (1.19,1.95)		1.50 (1.12,2.01)		1.95 (1.59,2.38)		1.35 (1.07,1.71)	
Unknown ^b^	na											
CT diagnosis		<0.01				0.86				0.17		
vCT (rCT not tested)	1				1				1			
vCT (rCT negative)	1.41 (1.08,1.84)				1.10 (0.68,1.78)				1.38 (0.90,2.11)			
vCT & rCT	1.35 (1.18,1.54)				0.92 (0.73,1.16)				1.17 (0.96,1.43)			
rCT (vCT negative)	1.50 (1.16,1.93)				0.98 (0.62,1.54)				1.32 (0.89,1.97)			
No. of sex partners		0.01				0.51				0.65		
0 or 1	0.97 (0.83,1.14)				1.06 (0.80,1.40)				1.02 (0.80,1.31)			
2 or 3	0.82 (0.72,0.94)				1.09 (0.86,1.38)				0.92 (0.75,1.14)			
≥ 4	1				1				1			
Unknown ^b^												

aOR: adjusted odds ratio; CI: confidence interval; OR: odds ratio; *P*: two-sided *P* rCT: rectal *Chlamydia trachomatis*, vCT: vaginal *Chlamydia trachomatis*

^a^ target population includes women 18 years or older with vaginal or rectal CT and negative for HIV, syphilis, and *Neisseria gonorrhoeae*, during the study inclusion period: Apr. 2016–Sept. 2017

^b^ unknown category was included in the models (not presented)

#### Associated factors for noninclusion from preselected women

In the multivariate analyses (Table 3), factors independently associated with noninclusion in the preselected women were study site Amsterdam (compared to South Limburg), non-Western migration background, no STI history, being 18–20 years of age (compared to 24 years or older) and a high educational level (rather than a low educational level). A medium educational level (compared to low education) was inversely associated.

#### Associated factors for noninclusion from the target population

In the multivariate analyses (Table 3), factors independently associated with noninclusion were study site Amsterdam (compared to South Limburg), non-Western migration background, no STI history, and a high educational level (compared to low education). A medium educational level (compared to low educational level) was inversely associated.

### Factors associated with attrition risk

The attrition risk was higher in women with low educational level (compared to high educational level) and in women aged 18–20 years (compared to 24 years or older) (Table 4).

**Table 4:** Attrition (i.e., patients lost to follow-up or withdrawn) rates and Hazard Ratios, FemCure during the 3-month follow-up period (Apr. 2016–Dec. 2017).

	Attrition events	Person time at risk	Incidence rate	C		C	
	n	Months	n/100 person months	HR (95% CI)	*P*	aHR (95% CI)	*P*
Study site					0.25		
South Limburg	45	444.60	10.12 (7.38,13.54)	1			
Amsterdam	41	454.44	9.02 (6.47,12.24)	0.91 (0.60,1.39)			
Rotterdam	47	343.32	13.69 (10.06,18.21)	1.28 (0.85,1.93)			
Age (in years)					<0.01		0.03
18–20	57	319.20	17.86 (13.53,23.14)	2.15 (1.40,3.29)		1.71 (1.06,2.76)	
21–23	43	463.92	9.27 (6.71,12.49)	1.25 (0.79,1.97)		1.08 (0.67,1.75)	
≥24	33	459.24	7.19 (4.95,10.09)	1		1	
Background					0.04		
Western	117	1162.08	10.07 (8.33,12.07)	1			
Non-Western migrant	16	80.28	19.94 (11.39,32.37)	1.71 (1.01,2.88)			
Education					<0.01		<0.01
Low	68	405.48	16.77 (13.02,21.26)	2.90 (1.73,4.88)		2.36 (1.35,4.14)	
Middle	47	463.20	10.15 (7.46,13.49)	1.92 (1.12,3.31)		1.48 (0.80,2.72)	
High	18	370.92	4.85 (2.88,7.67)	1		1	
Unknown ^a^	0	2.76	na	na		Na	
STI history					0.74		
Yes	41	360.12	11.38 (8.47,12.91)	1			
No	91	865.44	10.52 (7.42,15.45)	0.94 (0.65,1.36)			
Unknown ^a^	1	16.80	na	na			
Anatomic site CT diagnosed					0.47		
vCT (rCT not tested)	87	770.88	11.29 (9.04,13.92)	1			
vCT (rCT negative)	6	55.92	10.74 (3.94,23.35)	0.97 (0.42,2.21)			
vCT & rCT	37	341.04	10.85 (7.64,10.85)	0.96 (0.66,1.42)			
rCT (vCT negative)	3	74.52	4.03 (0.83,11.77)	0.40 (0.13,1.28)			
No. of sex partners					0.11		
0 or 1	31	278.04	11.15 (7.58,15.83)	1.42 (0.86,2.35)			
2 or 3	73	569.64	12.82 (10.05,16.11))	1.59 (10.3,2.44)			
≥ 4	29	389.16	7.45 (4.99,10.70)	1			
unknown ^a^	0	5.52	na	na			

aHR: adjusted Hazard Ratio; CI: confidence interval; HR: Hazard Ratio; Na: not assessed due to low numbers in the “unknown” category; *P*: two-sided *P*; rCT: rectal *Chlamydia trachomatis*, vCT: vaginal *Chlamydia trachomatis*

^a^ not included in the multivariate model due to little person time in the unknown categories

### Study evaluation

The responses on the study evaluation questionnaires in women who were retained in the study showed a high study satisfaction (Table 5).

**Table 5:** End of follow-up study evaluation responses from the retained participants in the prospective multicenter FemCure cohort study on *Chlamydia trachomatis* in women.

	n/N = 426^ (%)
Experience with the study	
Study instructions face-to-face by study nurse: (very) clear	424 (99.5)
Study instructions by website: (very) clear	318 (74.6)
Study instructions by booklet: (very) clear	407 (95.5)
Sampling: (very) easy	408 (95.8)
Communication (e.g., text messaging, email, phone): (very) satisfied	407 (95.5)
Study clinic visits: (very) satisfied	416 (97.7)
Overall experience with study: (very) good	411 (96.5)
Main reasons for participation	
Contribute to more knowledge on rectal chlamydia	327 (76.8)
I could have rectal chlamydia even without anal sex	135 (31.7)
To help others	294 (69.0)
At the end of the study (week 12), to receive a chlamydia test with result and treatment (when needed)	35 (8.2)
Monetary incentive	34 (8.0)
Awareness of rectal chlamydia	
Heard of rectal chlamydia before the study	186 (43.7)
(completely) agree that is important for me to talk about rectal chlamydia:	
… with friends/sex partners	349 (81.9)
… with my general practitioner or STI clinic nurse	297 (69.7)

^427 participants were retained in the study, 1 had missing evaluation data; ~measured on a 5-point Likert scale and dichotomized for analyses (categories 4 [agree] and 5 [completely agree]—shown in the table, versus categories 1–3)

## Discussion

We demonstrated that chlamydia-infected women can be included and retained in an intensive prospective cohort with 3 months of follow-up. The FemCure study was set up to determine risks for outcomes during follow-up in a group of CT infected women. Here, we assessed the external validity of the FemCure study, and demonstrated that the included population was not fully representative of all STI clinic women. Due to sampling bias, women who had a high educational level, no STI history, or with a non-Western migrant background, were underrepresented. We also assessed the internal validity, and concluded that the number of retained women was sufficient for the planned analyses, but young women and less educated women were more difficult to retain. This selective attrition may have introduced potential selection bias, which will be taken into account when interpreting the FemCure study’s findings.

To aid other researchers, we added a description of the strategies employed to engage women into the study: strategies shown by previous studies to be helpful to improve inclusion and retention (Table 1). A limitation of the current evaluation is that we could not assess the impact of the separate strategies as we did not have a control group and all strategies were simultaneously implemented.

To be included, women first had to be preselected, which was restricted (budgetary reasons) by the predefined research capacity to handle inclusions. Among the preselected women, 31% were included, which was lower than in other prospective studies in the area of sexual health that involved women, applying self-collection of samples at home [32–34]. In FemCure, not being able to attend the scheduled clinic visits or expecting too much effort to complete the study were important reasons for noninclusion. Among the included women, 76% completed the study, which was substantial and comparable to other prospective sexual health studies in women [32–35].

The design of our study was aimed at high internal validity [1], following a large group of women over 3 months to assess the risks for study outcomes (CT detection) after treatment. Thereby, we were interested in including a diverse sample of women from different subsets, to be able to adjust risks (confounding) and exploring heterogeneity of risks (effect modification).

In our sample, some subgroups participated less often due to exclusion criteria and self-selection, introducing sampling bias. The FemCure study sample underrepresented women with a high educational level, women without STI history, and non-Western migrant women. Women with full-time day jobs may not have had time to attend the clinic for required study visits, contributing to the underrepresentation of highly educated women. CT-infected women without an STI history possibly were likely less motivated to participate because of a lower health problem awareness than the previously infected women. As a substantial number of women with high educational level and women without STI history participated, statistical adjustment for these variables will be possible. To be included, women should be able and willing to free their time for the required clinic attendances and should understand the Dutch language. Likely, non-Western migrant women faced language barriers to participation. The small number of non-Western migrant women included in the study population hinders statistical adjustment for this characteristic. The diversity of our study population was, thus, confined by self-selection and by exclusion criteria, such as very young (<18 years) age (ethical reasons), recent antibiotics use, or STI co-infection (potential confounders that were expectedly too infrequent to statistically adjust for). The study nurses’ availability was fairly random and thereby the restricted capacity to handle inclusions presumably did not lead to the exclusion of specific subgroups. It should be noted that selective participation of any subset does not bias the risk estimates obtained in a prospective study, as the outcomes have not yet occurred at inclusion (they occur during follow-up) [1,14]. Further, statistical inferences to excluded subsets will not be possible, although the risks may be applicable to such subsets [14].

Importantly, the lower participation at the Amsterdam clinic resulted from the predefined target number of inclusions that were similar for each clinic, while the Amsterdam clinic served a much larger number of clients (large denominator). Further, young women were generally not underrepresented as young (18–20 years of age) women were included less often but more often preselected.

The sampling bias regarding education, no STI history, and non-Western migrant background resulted in a sample that was not completely representative of the target population. Even though data from the clinic-based cohorts are considered more representative of the “real world” than data from the randomized trials with numerous exclusion criteria [36], caution should be taken when making population inferences from the frequency distributions of FemCure’s baseline characteristics. Even with a representative study sample, inferring population impact can be a tricky undertaking as populations tend to change over time, such as STI clinic populations that vary in composition due to policy changes. Correspondingly, STI clinic populations may not represent the general population.

Our target number of women with retention was reached, ensuring sufficient power for the planned analyses [15]. Retained participants expressed a high study satisfaction and it is notable that nearly all (95%) of the 452 women who completed the first follow-up measurement were retained until the end of the study. However, young women and women with lower educational level were more difficult to retain; they tended to directly drop out after the initial inclusion contact, as was observed in previous studies [32]. Attrition does not necessarily invalidate the study as a sufficient number of women was still retained. However, selective attrition may affect the internal validity when the reasons for stopping data collection were related to both exposure and outcome [1,8]. This may introduce selection bias and lead to an underestimation or overestimation of the risk estimates. We will examine the education and age heterogeneity of risks by testing for effect modification, e.g., assess whether the association between the treatment type and post-treatment CT detection differs by educational level. Potentially, we may consider applying probability-of-exposure weights or inverse-probability-of-attrition weights to compensate for possible under-or-over estimation of risks [37].

By simultaneously implementing a mix of strategies, we aimed to optimize participation. We highlight a few previously found effective components [23–34] that in our practical experience were viewed as critical for the success of this study. Training and team building have resulted in a highly functioning and highly motivated interdisciplinary research team. Sufficient staffing was a challenge with the restricted budgetary funds and was extra invested in by the clinics. All nurses were women who at that time worked at the STI clinic in patient care and were experienced in addressing young women with various backgrounds on sexual health topics in a nonjudgmental way. They were committed to the protocol execution and patient follow-ups. Patient monitoring, reminders, and data handling were managed by a logistical team and a well-built computer program. The research was embedded in an existing clinical infrastructure providing a trusted and familiar research environment for patients. The health problem that this study dealt with affected the women themselves. Women received small monetary incentives and an additional STI checkup at week 12. Data collection was made easy with collection at home or at a nearby clinic and with clear instructions. A strategy that proved helpful in maintaining contact was text messaging. All women received a reminder a day before each data collection and when they did not fill in a questionnaire. Out of all women who were retained in the study 65% (n = 278/427) had received at least one such reminder. This proportion was 70% in retained women with a low educational level and 70% in women aged 18–20 years. A close-contact environment was further enabled by personalized contact with participants. Contact with study site staff was maintained by regular feedback from the coordinating site, expressing gratitude, and addressing any issues that arose regarding the study processes.

To conclude, it is feasible to include and retain CT-infected women in an intensive prospective cohort in busy clinical settings with sufficient investment in the design and study infrastructure and use multifaceted strategies to maximize participation. Highly educated women, women with non-Western background, and women without STI history were less likely to participate, while young women and women with low educational level were more difficult to retain.

## List of abbreviations

aHR: adjusted Hazard Ratio
aOR: adjusted odds ratio
CI: confidence interval
CT: *Chlamydia trachomatis*
HR: Hazard Ratio
PM: person months
OR: odds ratio
rCT: rectal *Chlamydia trachomatis*
STI: sexually transmitted infection
vCT: vaginal *Chlamydia trachomatis*

## References

[1] Rothman KJ, Greenland S, Lash TL. Modern Epidemiology. 3^rd^ ed. Lippincott Williams & Wilkins; 2012

[2] Sedgewick P. Prospective cohort studies: advantages and disadvantages BMJ 2013;347: f6726

[3] NIH Guidelines on the inclusion of women and minorities as subjects in clinical research. Fed. Reg. 1994; 59:14508–14513

[4] Kim AM, Tingen CM, Woodruff TK. Sex bias in trials and treatment must end. Nature 2010; 465(7299):688–94 10.1038/465688a 20535184

[5] Geller SE, Koch AR, Roesch P, et al The More Things Change, the More They Stay the Same: A Study to Evaluate Compliance with Inclusion and Assessment of Women and Minorities in Randomized Controlled Trials. Acad Med. 2017 10 19 10.1097/ACM.0000000000002027 29053489 PMC5908758

[6] Dumas–Mallet E, Button KS, Boraud T, et al Low statistical power in biomedical science: a review of three human research domains. R Soc Open Sci. 2017 2 1;4(2):160254 10.1098/rsos.160254 eCollection 2017 Feb 28386409 PMC5367316

[7] Patil P, Peng RD, Leek JT. What Should Researchers Expect when They Replicate Studies? A Statistical View of Replicability in Psychological. Perspect Psychol Sci. 2016 7;11(4):539–44. 10.1177/1745691616646366 Science 27474140 PMC4968573

[8] Howe CJ, Cole SR, Lau B, et al Selection Bias Due to Loss to Follow Up in Cohort Studies. Epidemiology. 2016 1;27(1):91–7. 10.1097/EDE.0000000000000409 26484424 PMC5008911

[9] Elwood JM. Commentary: On representativeness. Int J Epidemiol. 2013 8 1;42(4):1014–1015. 10.1093/ije/dyt101 24062288

[10] Rothman KJ, Gallacher J, Hatch EE. Why representativeness should be avoided. Int J Epidemiol. 2013;42:1012–14 10.1093/ije/dys223 24062287 PMC3888189

[11] Rothman KJ, Gallacher JE, Hatch EE. Rebuttal: When it comes to scientific inference, sometimes a cigar is just a cigar. Int J Epidemiol. 2013 8;42(4):1026–8. 10.1093/ije/dyt124 24062292 PMC3781006

[12] Pizzi C, De Stavola B, Merletti F et al Sample selection and validity of exposure–disease association estimates in cohort studies. J Epidemiol Community Health. 2011 5; 65(5):407–11 10.1136/jech.2009.107185 20881022

[13] http://www.equator-network.org/library/ (last accessed Aug. 2018)

[14] Toerien M, Brookes ST, Metcalfe C, et al A review of reporting of participant recruitment and retention in RCTs in six major journals. Trials. 2009 7 10; 10:52 10.1186/1745-6215-10-52 PMC2717957 19591685

[15] Dukers–Muijrers NH, Wolffs PF, Eppings L, et al Design of the FemCure study: prospective multicentre study on the transmission of genital and extra-genital Chlamydia trachomatis infections in women receiving routine care. BMC Infect Dis. 2016 8 8;16:381 10.1186/s12879-016-1721-x 27502928 PMC4977887

[16] Unemo M, Bradshaw CS, Hocking JS, et al Sexually transmitted infections: challenges ahead. Lancet Infect Dis. 2017 8;17(8):e235–e279. 10.1016/S1473-3099(17)30310-9 Epub 2017 Jul 9 28701272

[17] Senior K. Chlamydia: a much underestimated STI. Lancet Infect Dis. 2012 7;12(7):517–8 10.1016/s1473-3099(12)70161-5 22930827

[18] Haggerty CL, Gottlieb SL, Taylor BD, et al Risk of sequelae after *Chlamydia trachomatis* genital infection in women. J Infect Dis. 2010 6 15;201 Suppl 2:S134–55. 10.1086/652395 Review 20470050

[19] Lanjouw E, Ouburg S, de Vries HJ, et al 2015 European guideline on the management of *Chlamydia trachomatis* infections. Int J STD AIDS. 2016 4;27(5):333–48. 10.1177/0956462415618837 Epub 2015 Nov 24 26608577

[20] Geisler WM. Diagnosis and Management of Uncomplicated *Chlamydia trachomatis* Infections in Adolescents and Adults: Summary of Evidence Reviewed for the 2015 Centers for Disease Control and Prevention Sexually Transmitted Diseases Treatment Guidelines. Clin Infect Dis. 2015 12 15;61 Suppl 8:S774–84. 10.1093/cid/civ694 26602617

[21] Dukers–Muijrers NH, Schachter J, van Liere GA, et al What is needed to guide testing for anorectal and pharyngeal *Chlamydia trachomatis* and *Neisseria gonorrhoeae* in women and men? Evidence and opinion. BMC Infect Dis. 2015 11 17;15:533 10.1186/s12879-015-1280-6 26576538 PMC4650297

[22] Visser M, van Aar F, Op de Coul ELM, et al. Sexually transmitted infections in the Netherlands in 2017. 10.21945/RIVM-2018-0012 PMC5574382 28159917

[23] Campbell MK, Snowdon C, Francis D, et al Recruitment to randomised trials: strategies for trial enrollment and participation study. The STEPS study. Health Technol Assess. 2007 11;11(48):iii, ix–105. 10.3310/hta11480 17999843

[24] Wiemann CM, Chacko MR, Tucker JC, et al Enhancing recruitment and retention of minority young women in community-based clinical research. J Pediatr Adolesc Gynecol. 2005 12;18(6):403–7 10.1016/j.jpag.2005.09.006 16338606

[25] Caldwell PH, Hamilton S, Tan A, et al Strategies for increasing recruitment to randomised controlled trials: systematic review. PLoS Med. 2010 11 9;7(11):e1000368 10.1371/journal.pmed.1000368 21085696 PMC2976724

[26] Fletcher B, Gheorghe A, Moore D, et al Improving the recruitment activity of clinicians in randomised controlled trials: a systematic review. BMJ Open. 2012 1 6;2(1):e000496 10.1136/bmjopen-2011-000496 Print 2012 22228729 PMC3253423

[27] Treweek S, Pitkethly M, Cook J, et al Strategies to improve recruitment to randomised trials. Cochrane Database Syst Rev. 2018 2 22;2:MR000013. 10.1002/14651858.MR000013.pub6 29468635 PMC7078793

[28] Brueton VC, Tierney J, Stenning S, et al Strategies to improve retention in randomised trials. Cochrane Database Syst Rev. 2013 12 3;(12):MR000032. 10.1002/14651858.MR000032.pub2 24297482 PMC4470347

[29] Barnett J, Aguilar S, Brittner M, et al Recruiting and retaining low-income, multi-ethnic women into randomized controlled trials: successful strategies and staffing. Contemp Clin Trials. 2012 9;33(5):925–32. 10.1016/j.cct.2012.06.005 Epub 2012 Jun 23 22732312 PMC3430447

[30] Andrighetti HJ, Semaka A, Austin JC. Women's experiences of participating in a prospective, longitudinal postpartum depression study: insights for perinatal mental health researchers. Arch Womens Ment Health. 2017 8;20(4):547–559. 10.1007/s00737-017-0744-7 Epub 2017 Jun 10 28600644 PMC5511519

[31] Daniels LA, Wilson JL, Mallan KM, et al Recruiting and engaging new mothers in nutrition research studies: lessons from the Australian NOURISH randomised controlled trial. Int J Behav Nutr Phys Act. 2012; 9: 129 10.1186/1479-5868-9-129 (https://www.ncbi.nlm.nih.gov/pmc/articles/PMC3507820/) 23107387 PMC3507820

[32] Walker J, Fairley CK, Urban E, et al Maximising retention in a longitudinal study of genital *Chlamydia trachomatis* among young women in Australia. BMC Public Health. 2011 3 9;11:156 10.1186/1471-2458-11-156 21385471 PMC3061916

[33] Forcey DS, Walker SM, Vodstrcil LA, et al Factors associated with participation and attrition in a longitudinal study of bacterial vaginosis in Australian women who have sex with women. PLoS One. 2014 11 20;9(11):e113452 10.1371/journal.pone.0113452 eCollection 2014 25412421 PMC4239064

[34] Trent M, Chung SE, Gaydos C, et al Recruitment of Minority Adolescents and Young Adults into Randomised Clinical Trials: Testing the Design of the Technology Enhanced Community Health Nursing (TECH-N) Pelvic Inflammatory Disease Trial. Eur Med J Reprod Health. 2016 8;2(1):41–51 27617108 PMC5013541

[35] Kristman V, Manno M, Côté P. Loss to follow-up in cohort studies: how much is too much? Eur J Epidemiol. 2004;19(8):751–60. 10.1023/b:ejep.0000036568.02655.f8 15469032

[36] Geng EH, Glidden DV, Bangsberg DR, et al A causal framework for understanding the effect of losses to follow-up on epidemiologic analyses in clinic-based cohorts: the case of HIV-infected patients on antiretroviral therapy in Africa. Am J Epidemiol. 2012 5 15;175(10):1080–7. 10.1093/aje/kwr444 Epub 2012 Feb 3 22306557 PMC3353135

[37] Weuve J, Tchetgen EJ, et al Accounting for bias due to selective attrition: the example of smoking and cognitive decline. Epidemiology. 2012 1;23(1):119–28. 10.1097/EDE.0b013e318230e861 21989136 PMC3237815

